# Effects of different sterilization methods of herbal formula on phytochemical compounds and antibacterial activity against mastitis-causing bacteria

**DOI:** 10.14202/vetworld.2020.1187-1192

**Published:** 2020-06-25

**Authors:** Dian Wahyu Harjanti, Fajar Wahyono, Vincentia Rizke Ciptaningtyas

**Affiliations:** 1 Department of Animal Science, Faculty of Animal and Agricultural Sciences, Diponegoro University, Semarang, Central Java, Indonesia; 2 Department of Clinical Microbiology, Faculty of Medicine, Diponegoro University, Semarang, Central Java, Indonesia

**Keywords:** herbal extract, mastitis, phytobiotic, phytochemical compound, sterilization method

## Abstract

**Background and Aim:**

The current phytobiotic industry is struggling to determine a proper sterilization method for an herbal formula that comprises multiple plant extracts. Hence, this study aims to investigate the effects of two sterilization methods of herbal formula on phytochemical compounds and antibacterial activity against mastitis-causing bacterial isolates.

**Materials and Methods:**

The herbal formula comprised the extracts of *Piper betle* leaves, *Curcuma domestica*, and *Curcuma zanthorriza*. We applied two sterilization methods – sterilization using 0.45 μM syringe filter and sterilization using an autoclave at 121°C for 15 min. After sterilization, we measured phytochemical contents (phenol and flavonoid) of the herbal formula. Using the disk diffusion method, the antibacterial susceptibility test of the sterilized herbal formula against mastitis-causing bacterial isolates was conducted. Tetracycline, erythromycin, and penicillin – common antibiotics for mastitis therapy in dairy farms – were used as standard antibiotics.

**Results:**

Compared with autoclave sterilization, syringe filter sterilization resulted in less (p<0.05) phenolic and flavonoid contents. Against *Escherichia coli* and *Staphylococcus aureus*, the filter sterilized herbal formula (*E. coli*: 65.9%-73%; *S. aureus*: 6.2%-18.1%) markedly reduced the antibacterial activity than the autoclave-sterilized herbal formula (*E. coli*: 2.1%-3%; *S. aureus*: 4.5%-10.7%).

**Conclusion:**

This study establishes that autoclave sterilization of the herbal formula is the best sterilization method that exerts minimal adverse effects on the phytochemical compounds and could sustain the antibacterial efficacy against mastitis-causing bacteria. Hence, the herbal formula could be used as an alternative treatment for bovine mastitis.

## Introduction

Antibiotic residues in raw milk and milk products are a global health hazard. Recent years have witnessed several countries around the world implementing extensive bans on the use of antibiotics for livestock animals. Accordingly, the antimicrobial effects of plants have been extensively explored as an alternative to synthetic antibiotics. Ethnoveterinary medicine, which includes the use of medicinal plants, is regarded as an alternative to treat bovine mastitis, as well as prevent microbial resistance and antibiotic residues in milk. Recent research has demonstrated the efficacy of some herbs with antimicrobial, antifungal, anti-inflammation, and immunoreactive properties [1-4]. Research on dairy cows using herbs as feed additives reported a significant decline in the somatic cell count in milk, suggesting healing of inflammation in the mammary glands [5-9]. However, dietary herbal feed additive alone is insufficient to cure mammary inflammation in mastitis cases. To date, antibiotics remain the most effective method of curing mastitis.

Some preclinical studies determined the antimicrobial activities of herbs using several bioassays such as disk diffusion, well diffusion, broth agar, and agar dilution methods [10,11]. In addition, some previous studies successfully conducted the antibacterial susceptibility test of various single herbal extracts against mastitis-causing bacteria, which were isolated from mastitis milk [4]. The most persistent problem in preclinical studies is the contamination due to bacteria, fungus, and mold, which, perhaps, arises while executing stages of extraction and following experimental procedures. The standard method to sterilize herbal extract is filtrating using syringe microfiltration membrane [10]; however, the use of these membranes is energy consuming and expensive. Besides, preclinical studies further use of plant extract mixtures as herbal formulas in clinical studies and the commercialization of herbal antibiotics need sterile materials. In the pharmaceuticals industry, the safety of herbal medicines has been a concern since the commercialization of herbal products began. Regarding bovine mastitis, sterile antibiotic administration into the mammary gland through teat meatus (intramammary treatment) remains the most effective method to cure mastitis. Some widely applied sterilization methods in research and antibiotic manufacturing have been 0.45 μM syringe filter membrane and gamma irradiation [12-14]. Of note, a good sterilization method must effectively eliminate microbes without damaging the bioactive compound. Besides the antibacterial efficacy, the method should be cost-effective so that it could be applied in experiments and the pharmaceuticals industry.

This study aimed to investigate two different sterilization methods and compare the concentration of bioactive compounds and the antibacterial activity after sterilization. The antibacterial efficacy was tested against mastitis-causing bacteria, which was isolated from mastitis-infected cows. Notably, tetracycline, erythromycin, and penicillin, which are commonly used for mastitis therapy in dairy farms [4,15,16], were used as standard antibiotics in this study.

## Materials and Methods

### Ethical approval

Ethical approval was not necessary for this study.

### Study period and location

This study was conducted during the period April to August 2019. The experiment was conducted at Microbiology Laboratory, Medical Faculty Diponegoro University, Indonesia.

### Plant extraction and formulation

Plant materials were collected from Semarang regency, which is located in Central Java Province, Indonesia at 1200 m a.s.l. (above sea level). Plant materials were identified by Dr. Jumari, MSc. at Biology Department, Diponegoro University. All materials were dried and finely grounded into a powder. The maceration procedure for each material was conducted as described by Harjanti *et al*. [4]. *P. betle* leaves extract (PBE), *C. domestica* extract (CDE), and *C. zanthorriza* extract (CZE) obtained were formulated into the following three formulations: (i) Formula A (0.5 g PBE + 0.4 g CDE + 0.3 g CZE); (ii) formula B (0.6 g PBE + 0.3 g CDE + 0.3 g CZE); and (iii) formula C (0.7 g PBE + 0.2 g CDE + 0.3 g CZE). Then, each formula was added with 1.2 mL polyethylene glycol, 1 mL glycerin, and 60 mL sterile aquadest on a hot plate magnetic stirrer at 60°C with the rotational speed of 500 rpm. After 20 min, the mixtures were filtered using a filter paper (Whatman No. 41; Merck, Germany) into a sterile bottle and place the lid securely.

### Sterilization method

We applied two sterilization methods – sterilization using 0.45 μM sterile syringe filter (Axiva Biotech, India) and sterilization using an autoclave (HVE-50; Hirayama, Japan). Of note, filtration was performed using 0.45 μM sterile syringe filter membrane, and the flow through was collected in a sterile bottle. For autoclaving, the extract formula was autoclaved at 121°C for 15 min. The prepared sterile extracts were stored at 4°C before testing.

### Mastitis bacteria preparation

Gram-positive (*Staphylococcus aureus*) and Gram-negative (*Escherichia coli*) bacteria were isolated from mastitis-infected cows, which were reared in the local dairy farms in Central Java Province. The isolation procedure was performed as described by Harjanti *et al*. [17]. The bacteria were subcultured on house prepared 5% sheep blood agar (Oxoid™ Blood Agar Base CM0055).

### Antibacterial susceptibility testing

We used 0.85% NaCl to dilute the isolated colonies of *S. aureus* and *E. coli* until McFarland 0.5 turbidity was reached. Then, the solution of bacterial inoculum was swabbed onto Mueller-Hinton agar (MHA) plates (Oxoid™ Blood Agar Base CM0337). Next, a sterile blank paper disk (Oxoid™ CT0998B) was aseptically placed onto MHA. Then, 25 mL of three tested extract formulas were transferred to each disk. For positive control, 30 mg tetracycline (Oxoid™ CT0043B), 15 mg erythromycin (Oxoid™ CT0020), and 10 U penicillin G (Oxoid™ CT0043B) were used. The plates were incubated at 37°C for 24 h. We measured the inhibition zone diameter with a ruler in mm, using the unaided eye with a black background as a contrast.

### Total flavonoid and phenolic compounds

Using the aluminum chloride colorimetric method, we determined the total flavonoid content of each sterile extract formula, as described by Bag *et al*. [18]. Next, the total phenolics of each sterile extract formula were determined using the Folin–Ciocalteu reagent, as described by Ghasemzadeh *et al*. [19].

### Statistical analysis

In this study, a completely randomized design with three replicates was used to compare the antibacterial affectivity of three formulas, as well as compare the different sterilization methods. In addition, means comparison was performed using Duncan’s new multiple range test (p<0.05). All statistical analyses were performed using SPSS version 9.1 (SPSS Inc, Chicago, Illinois, USA).

## Results

### Phytochemical compounds of the herbal formula

**Figure-1:**
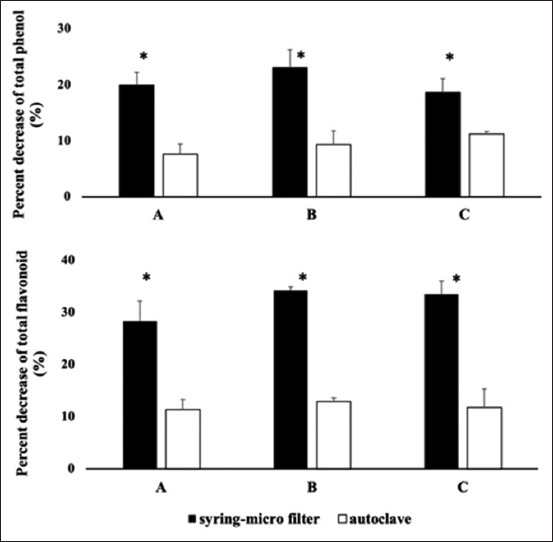
Comparison of percentage decrease of phenol and flavonoid concentrations after sterilization with syringe filter (▄) and autoclave (□). *(p<0.05). Sterilization methods applied were sterilization using syringe filter 0.45 μM and sterilization using autoclave at 121°C for 15 min

Tables-1 and 2 present the effects of different sterilization methods on the concentrations of phenol and flavonoid in the herbal formula. Table-1 shows that total phenol in formulas A–C sterilized using an autoclave did not differ markedly with those without sterilization (control). However, syringe filter sterilization for formulas A and C resulted in less phenolic concentration (p<0.05) than control and autoclave-sterilized formula. In addition, filter sterilized formula B exhibited a numerically lower concentration of phenol compared with control and autoclave-sterilized formula. Regarding flavonoid, both sterilization methods deceased the flavonoid concentration in the herbal formula (Table-2); however, the reduction was far less with autoclaving. The flavonoid concentration in autoclave-sterilized formulas A–C was higher (p<0.05) than those sterilized with the syringe filter. Moreover, the percentage decrease of phenol and flavonoid concentrations in the herbal formula after autoclave sterilization was less (p<0.05) than those sterilized using the syringe filter (Figure-1). The reduction of total phenol ranged 7.7%-11.2% using autoclave sterilization and 18.6%-23.1% using syringe filter sterilization. For total flavonoid, ­however, the reduction ranged 11.4%-13.0% with autoclave sterilization and 28.4%-34.3% with syringe filter sterilization.

**Table-1:** Phenol concentration (ppm) of herbal formula after sterilization with different method.

Herbal formula	Sterilization method		

	Extract without sterilization (control)	Extract sterilized with syringe filter	Extract sterilized with autoclave
Formula A	2325.4±112.7^a^	1858.7±38.9^b^	2144.7±63.0^a^
Formula B	2536.2±308.4	1940.3±179.3	2291.6±229.8
Formula C	3021.6±177.0^a^	2452.4±70.7^b^	2682.5±145.0^ab^

Values are mean±SEM of triplicate tests;

ab different superscripts in the same row are significantly different (p<0.05); sterilization methods applied were sterilization using syringe filter 0.45 μM and sterilization using autoclave at 121°C for 15 min

**Table-2:** Flavonoid concentration (ppm) of herbal formula after sterilization with different method.

Herbal formula	Sterilization method		

	Extract without sterilization (control)	Extract sterilized with syringe filter	Extract sterilized with autoclave
Formula A	819.4±13.5^a^	586.2±26.0^c^	725.8±16.5^b^
Formula B	509.8±2.5^a^	335.1±4.9^c^	443.8±3.2^b^
Formula C	523.7±19.7^a^	348.3±24.1^c^	461.1±4.3^b^

Values are mean±SEM of triplicate tests;

abc different superscripts in the same row are significantly different (p<0.05); sterilization methods applied were sterilization using syringe filter 0.45 μM and sterilization using autoclave at 121°C for 15 min

### Antibacterial susceptibility test

**Figure-2:**
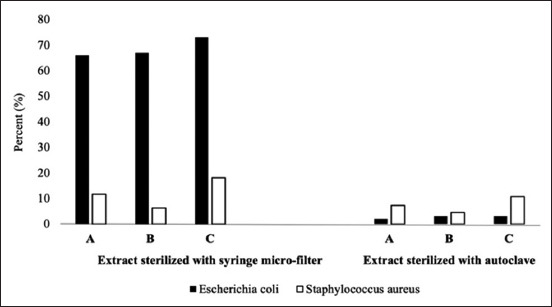
Comparison effect of different sterilization method on the percentage decrease of antibacterial activity against *Escherichia coli* (▄) and *Staphylococcus aureus* (□). Sterilization methods applied were sterilization using syringe filter 0.45 μM and sterilization using autoclave at 121°C for 15 min.

The antibacterial susceptibility tests were conducted for all herbal formulas against mastitis-causing bacterial isolates – *E. coli* and *S. aureus* (Table-3). The inhibitory zone’s diameter against *E. coli* for formulas A–C without sterilization was 9.7, 10, and 10 mm, respectively. In addition, we observed a variation in antibacterial activities of formulas A–C against *S. aureus* with diameter inhibitory zones at 22.3, 22.7, and 23.2 mm, respectively. Of note, the use of filter sterilized herbal formula reduced the antibacterial activity against *E. coli*, compared with control (without sterilization). The reduction of the antibacterial activity against *E. coli* was 65.9%-73% for filter sterilized herbal formula (Figure-2). Remarkably, autoclaving the herbal formula resulted in a low reduction of the antibacterial activity (2.1%-3%) compared with the control group. The antibacterial activity against *S. aureus* reduced to 6.2%-18.1% for filter sterilized formula and 4.5%-10.7% for autoclave-sterilized herbal formula, compared with the control group. In this study, penicillin was resistant to *E. coli*, whereas erythromycin and tetracycline were more susceptible. Regarding *S. aureus*, the inhibition zones obtained by all herbal formulas tested in this study were comparable with all standard antibiotics. Furthermore, the inhibition zones of standard antibiotics, tetracycline, erythromycin, and penicillin, for *S. aureus* were 22.7, 28.1, and 21.1 mm, respectively.

**Table-3:** Antibacterial activity of herbal formula against mastitis bacteria in comparison with standard antibiotic.

Sterilization Method	Herbal Formula	Mean inhibition zones (mm) of microorganism	

		*Escherichia coli*	*Staphylococcus aureus*
Extract without sterilization (control)	A	9.7	22.3
	B	10.0	22.7
	C	10.0	23.2
Extract sterilized with syringe filter	A	3.3	19.7
	B	3.3	21.3
	C	2.7	19.0
Extract sterilized with autoclave	A	9.5	20.7
	B	9.7	21.7
	C	9.7	20.7
Standard antibiotic	TE-30	23.8	22.7
	E-15	12.2	28.1
	P-10	1.0	21.1

Sterilization methods applied were sterilization using syringe filter 0.45 μM and sterilization using autoclave at 121°C for 15 min

## Discussion

Plants have a significant ability to synthesize secondary metabolites, which have recently been referred to as phytochemicals. Phenol and flavonoid are phytochemical compounds, which are naturally occurring and biologically active plant compound that has potential disease-inhibiting capabilities. In addition, phenol and flavonoid contents in plants reportedly exhibit antioxidant and antibacterial effects, as well as the immune system promoting and inflammatory effects [20]. Reportedly, phenol and flavonoid could be used to treat zoonotic diseases like mastitis [21-23]. Notably, understanding proper sterilization methods for preclinical research or clinical application is imperative to prevent huge damage and maximize the potential use of phytochemicals as an active compound in these herbs. Perhaps, thermal processing or sterilization method with high temperature could seriously damage the structure of bioactive compounds in extracts, which, in turn, decrease the antibacterial activity. Xu *et al*. [24] and Chaikham *et al*. [25] demonstrated that phytochemical compounds in herbal plants were decreased in quantity with thermal processing, and the reduction increased with increasing heating time. Likewise, a recent study illustrated that heat treatment had deleterious effects on phenolic concentrations of cauliflower and onion extracts [26]. Conversely, this study demonstrated that autoclave sterilization exerted a less negative impact on phenolic and flavonoid compounds, compared with the microfiltration method. To the best of our knowledge, no study has reported the effects of autoclave sterilization of herbal extract mixtures on the phytochemical compounds and antibacterial activity because it is believed that high temperature could adversely affect bioactive compounds. Indeed, several studies have used either a microfilter membrane or syringe microfilter membrane to sterilize herbal extracts [4,27-29]. Hashemi *et al*. [30] explored the effects of autoclaving various single aqueous extracts on the antibacterial activity. The difference with our study is that, for autoclaving, Hashemi *et al*. [30] impregnated the extract into a blank disk, which was then placed into a small glass bottle for sterilization using autoclave and only measured the antibacterial activity. We, however, prepared the mixed herbal formula and placed it into the bottle and then sterilized using an autoclave. The sterile formula packaged in the bottle is ready to use after sterilization. For the pharmaceutical industry, we believe that the method this study proposes is more applicable to phytoantibiotic manufacturing.

In the livestock industry, bovine mastitis plays a decisive role because of disturbing animal health, animal welfare, and causing substantial economic losses. Thus, antibiotic treatment is indispensable to maintain bovine udder health, animal welfare, and economic aspect in balance. Conversely, the emergence and spread of antibiotic resistance in milk is a pressing issue that could affect dairy product quality and human health. To date, several studies have explored the use of herbal remedies against bacterial isolates from mastitis-infected animals [6,7,16,21]. Reportedly, *S. aureus* and *E. coli* are the leading pathogens accountable for contagious mastitis in ruminants [16,17,31]. The effect of mastitis primarily depends on the etiology. The virulence factor influencing the bacteria pathogenicity in mastitis is the ability to form biofilm [32]. Bovine mastitis caused by both *S. aureus* and *E. coli* can range from being subclinical infection of the mammary gland to severe systemic disease [15,16]. Mastitis-causing *E. coli* bacteria are typically commensals [33]. Notably, *E. coli* incite strong inflammation through vigorous stimulation of cytokine synthesis, which, in turn, activate the local and general immune response of the host [34]. Nevertheless, evidence for the efficacy of antimicrobial treatment for *E. coli* mastitis is scarce [33]. Some previous studies suggested that *E. coli* isolates from mastitis have developed resistance to antimicrobials commonly used for dairy farms in years, including ampicillin, streptomycin, sulfonamides, and oxytetracycline [33,35,36]. This study reveals that *E. coli* was susceptible to erythromycin and tetracycline, but resistant to penicillin, whereas *S. aureus* was susceptible to all standard antibiotics (tetracycline, ­erythromycin, and penicillin). Likewise, this study demonstrated that *S. aureus*, *Streptococcus agalactiae*, and *Streptococcus dysgalactiae* are susceptible to penicillin and tetracycline, but *E. coli* is resistant to penicillin [35-37]. Some scholars recommend penicillin for the treatment of mastitis caused by Gram-positive pathogens [23,31,36,37]. Interestingly, in this study, all herbal formulas tested exhibited considerable growth-inhibiting activity against both *E. coli* and *S. aureus*, although the inhibition zones for Gram-negative *E. coli* were less than Gram-positive *S. aureus*. A similar pattern was observed in studies on bovine mastitis treatment using medicinal herbs [22,38,39]. Thus, some studies suggest that the difference in the susceptibility to antimicrobial agents between Gram-negative and Gram-positive bacteria could correlate with the composition and structure of the cytoplasmic membrane and cell wall of bacteria [34,40].

This study indicates the potential of the herbal extract to overcome both negative and positive bacterial infections and prevent resistance. The herbal formula possessed remarkable antibacterial activities because of the presence of phytochemical components, as biological or therapeutic activities of herbs closely correlate with their chemical components. In addition, *P. betle* leaves, *C. domestica*, and *C. zanthorriza* are medicinal plants that have been used as a folk medicine for a long time. Several studies have demonstrated that the antibacterial mechanism of phenolic compound correlates with their ability to modify the membrane permeability and affect the rigidity of cell wall, which induce the integrity losses [40,41]. Flavonoids possess a diverse range of pharmacological properties, including antibacterial and anti-inflammatory activities. The previous research has endeavored to determine whether the flavonoid activity is bacteriostatic or bactericidal by conducting time-kill studies and reported that flavonoid is not killing bacteria cells but merely inducing the formation of bacterial aggregates and thereby decreasing the bacteria number [41]. A recent study suggested that flavonoid did not inhibit bacterial growth but inhibited biofilm formation [42]. Reportedly, the biofilm formation is the pathogenicity virulence factor of bacteria [32].

## Conclusion

This study establishes that sterilization of the herbal formula using an autoclave at 121°C for 15 min is the best method that exerts a less negative effect on the phytochemical compounds and could maintain the antibacterial efficacy against mastitis-causing bacteria. Hence, the herbal formula could be used as an alternative treatment for bovine mastitis.

## Authors’ Contributions

DWH designed, conducted the experiment, and prepared the manuscript. FW conducted the herbal formulation experiment and revised the manuscript. VRC conducted the microbiological experiment, data analysis and revised the manuscript. All authors read and approved the final manuscript.
